# Improving nutritional quality, aroma profile and bioactive retention of rocket juice via thermosonication: a support vector regression-based optimization

**DOI:** 10.3389/fnut.2025.1721498

**Published:** 2026-01-07

**Authors:** Okan Levent, Mehmet Ali Şimşek, Seydi Yıkmış, Selinay Demirel, Melikenur Türkol, Nazan Tokatlı Demirok, Hatice Er, Moneera O. Aljobair, Emad Karrar, Nazlı Tokatlı, Isam A. Mohamed Ahmed

**Affiliations:** 1 Department of Food Engineering, Faculty of Engineering, Inonu University, Malatya, Türkiye; 2 Department of Computer Technologies, Vocational School of Technical Sciences, Tekirdag Namik Kemal University, Tekirdag, Türkiye; 3 Department of Food Technology, Tekirdag Namık Kemal University, Tekirdag, Türkiye; 4 Nutrition and Dietetics, Faculty of Health Sciences, Tekirdag Namık Kemal University, Tekirdag, Türkiye; 5 Corlu Vocational School, Tekirdag Namık Kemal University, Tekirdag, Türkiye; 6 Department of Sports Health, College of Sports Sciences and Physical Activity, Princess Nourah Bint Abdulrahman University, Riyadh, Saudi Arabia; 7 Department of Plant Sciences, North Dakota State University, Fargo, ND, United States; 8 Department of Computer Engineering, Faculty of Engineering and Natural Sciences, Istanbul Health and Technology University, Istanbul, Türkiye; 9 Department of Food Sciences and Nutrition, College of Food and Agricultural Sciences, King Saud University, Riyadh, Saudi Arabia

**Keywords:** bioactive compounds, *in vitro* digestion, machine learning, rocket juice, support vector regression, thermosonication

## Abstract

This study investigates the application of thermosonication (TS) to improve the functional properties of roka (*Eruca vesicaria subsp. sativia*) water. Processing parameters, including time (8–16 min), amplitude (60–100%), and temperature (40–60 °C), were optimised using a comparative approach combining the response surface method (RSM) and support vector regression (SVR). The total phenolic content (TPC) increased to 86.04 mg GAE/100 mL with TS, representing an 8.1% rise compared to the control group and an 18.3% increase over pasteurization. Likewise, the total chlorophyll level reached 16.98 mmol TE/L from 9.67 g/100 mL, and *β*-carotene rose to 24.90 mg/100 mL (*p* < 0.05). Pasteurization caused losses of 15–30% in these components. In the phenolic profile, significant increases were observed in chlorogenic acid (42.05 μg/mL), caffeic acid (15.66 μg/mL), and quercetin (4.28 μg/mL). A total of 31 compounds were identified in aroma analysis; with TS treatment, levels of 3-Hexen-1-ol (15.70 μg/kg) and 1-hexanol (2.01 μg/kg) were preserved or increased. In *in vitro* digestion tests, the TS group demonstrated the highest bioavailability, even during the intestinal phase. For example, RSM demonstrated high compliance coefficients (*R*^2^ = 0.99), while SVR showed strong predictive performance (CV *R*^2^ = 0.84), particularly for FRAP. Overall, the results suggest that thermosonication is an innovative method for protecting and enhancing bioactive compounds in rocket juice.

## 1 Introduction

Rocket (*Eruca vesicaria subsp. sativa*) is a member of the *Brassicaceae* family (1). It is an endemic, annual species that is characterized by a spicy, sharp taste (2). Rocket is abundant in biologically active compounds, including polyphenols, ascorbic acid, glucosinolates, carotenoids, and fibre (3). This is particularly apparent given its status as a widely favoured ingredient in fresh vegetables globally. Given its considerable commercial value, rocket can be regarded as a notable food crop (4). Rocket has been utilized within the domain of traditional medicine on account of its antimicrobial, astringent, laxative, rubefacient, digestive, diuretic, emollient, depurative, tonic, and stimulant properties (5).

Traditional thermal methods commonly used in food processing can compromise the stability of heat-sensitive ingredients, resulting in losses of aroma, color, and nutritional content. Thermosonication, by contrast, offers a promising alternative that minimizes these quality losses while effectively inactivating enzymes and microbes (6, 7). The demand for high-quality, additive-free foods is increasing. This has led to interest in advanced technology that can meet this demand while preserving food properties (8). Thermosonication (TS) is an innovative method that applies ultrasound waves at elevated temperatures and is considered an alternative technology in food processing (9). Thermosonication has been demonstrated to enhance the efficiency of enzyme and microbial inactivation, thereby minimising nutritional degradation and product quality deterioration. This is achieved by reducing the elevated temperatures and extended processing times conventionally required for heat treatments (10).

Response Surface Methodology (RSM) is a widely used statistical and mathematical optimization approach that allows multiple variables to be modeled and analyzed simultaneously (11). Since the quality attributes of thermosonicated rocket juice—such as FRAP capacity and total chlorophyll content are strongly influenced by parameters including processing time, temperature, and amplitude, an appropriate modelling strategy is essential. Therefore, in optimizing thermosonication conditions for rocket juice, RSM provides a systematic framework for identifying parameter interactions and determining the combination that yields the most desirable quality characteristics.

Support Vector Regression (SVR), a machine learning algorithm that can capture nonlinear patterns with strong generalization performance, is a machine learning method used in food science and technology as well as food processing (FPMO). Several studies have applied SVR in various contexts, for example, modelling the modification of bentonite for oil bleaching efficiency (12) or estimating stable isotope ratios in coffee to determine geographical origin (13). Its primary advantage lies in accurately modelling complex, nonlinear relationships that traditional statistical methods may miss. ML algorithms are utilized in food technology across various areas, including quality control, shelf-life prediction, modelling of sensory characteristics, and optimization of production processes. Algorithms such as ANN, SVM, RF, and SVR are particularly effective in modelling the complex and nonlinear dynamics of food processing (14, 15).

There are also studies in the literature that investigate which approach has higher accuracy and generalization ability in modelling and optimizing food processing data by comparing SVR with RSM (16). In this study, RSM and SVR analysis were employed to analyze rocket water. Although RSM is traditionally the most widely used method in experimental design, it is limited to nonlinear relationships. The literature shows that SVR is more successful than RSM, especially for small samples and complex relationships (17). The aim of our study is therefore to compare the classical RSM approach with the modern machine learning method of SVR, thereby highlighting the strengths and weaknesses of each.

The current literature lacks a comprehensive study on optimizing rocket juice components via thermosonication. Therefore, this study focuses on optimizing key thermosonication parameters—duration, temperature, and amplitude—to enhance the antioxidant capacity (FRAP) and total chlorophyll content of rocket juice, and to characterize its bioactive compounds, phenolic profile, and aroma components under optimal conditions. By doing so, the study aims to contribute to the development of a product with improved functional properties and higher quality, and to make an original contribution to the literature by demonstrating the potential of thermosonication as an alternative to traditional heat treatments, particularly for rocket juice.

## 2 Materials and methods

### 2.1 Materials

Rocket (*Eruca vesicaria subsp. sativa*) samples were supplied by local producers in the Tekirdağ region. During the preliminary analysis stage, the samples were refrigerated at +4 °C to preserve their biochemical integrity. During the preparation process, stems and mature plant parts were separated and removed. Homogenization was performed using a Waring brand commercial blender (Model HGB2WTS3) to ensure uniform particle size. The suspension was filtered through filter paper to remove cellulose. The sample was vortexed for a minute to standardise the macromolecular distribution. Rocket juice was used as the control group (C-RJ).

### 2.2 Methods

#### 2.2.1 Sample preparation and treatments

Rocket juice samples were prepared and processed using two methods. For thermal pasteurization, 100 mL of rocket juice was placed in glass bottles and pasteurized at 85 ± 1 °C for 2 min in a water bath (Wisd, model WUC-D06H, Daihan, Wonju, Korea). Following pasteurization, samples were allowed to reach ambient temperature and stored at −20 ± 1 °C until further analysis (P-RJ).

For thermosonication (TS), 100 mL of P-RJ samples were processed using an ultrasonic device (UP200S, Hielscher Ultrasonics, Berlin, Germany) at 26 kHz and 200 W. Different amplitude levels (60–100%), processing times (4–12 min), and temperatures (40–60 °C) were tested. An ice-water bath was used to maintain temperature control during processing, and TS-treated samples (TS-RJ) were rapidly cooled and stored at −18 ± 1 °C.

#### 2.2.2 Response surface methodology (RSM)

The effects of thermosonication on FRAP and total chlorophyll levels in rocket juice were evaluated using RSM and Minitab 18.1.1.15. Experimental points were designed within the scope of the optimisation (Table 1), and the model’s validity was confirmed using *R*^2^, adjusted *R*^2^, goodness-of-fit, and ANOVA results (Table 2). RSM was applied using a Box–Behnken design to optimize the processing conditions. The design included three independent factors, each at three levels (−1, 0, +1), resulting in 15 experimental runs (12 factorial points +3 centre-point replicates). The three centre-point repetitions were included to provide an estimate of pure experimental error and to test for lack of fit. In the study, processing time (X_1_), amplitude (X_2_), and temperature (X_3_) were considered independent variables, while FRAP and total chlorophyll were considered dependent variables. A second-degree polynomial equation (Equation 1) was used for modelling.


$$y=\beta_{0}+\sum_{i=1}^{3}\beta_{i}X_{i}+\sum_{i=1}^{3}\beta_{\mathrm{ii}}X_{i}^{2}+\sum_{\begin{array}{c} i=1\\ i<j \end{array}}^{3}\sum_{j=1}^{3}\beta_{\mathrm{ij}}X_{i}X_{j}$$ (1)

**Table 1:** Comparison of RSM and SVR prediction performance for FRAP/chlorophyll.

Run no.	Independent variables			Dependent variables					
	Time (X_1_) (min)	Amplitude (X_2_) (%)	Temperature (X_3_) °C	Total chlorophyll (g/100 mL)			FRAP (mmol TE/L)		
				Experimental data	RSM predicted	SVR predicted	Experimental data	RSM predicted	SVR predicted
1	16	60	50	7.08	6.98	7.07	13.16	13.05	13.17
2	12	60	40	8.74	8.84	8.75	14.78	14.96	14.79
3	12	60	60	9.21	9.28	9.22	14.10	14.37	14.11
4	16	80	60	7.15	7.20	7.16	13.29	13.21	13.30
5	8	100	50	6.98	7.09	7.10	12.96	13.12	12.97
6	8	80	60	8.77	8.76	8.78	14.29	14.34	14.30
7	16	100	50	7.47	7.52	7.48	14.40	14.69	14.41
8	12	80	50	9.67	9.67	9.66	16.48	16.51	16.47
9	12	80	50	9.67	9.67	9.66	16.48	16.51	16.47
10	8	60	50	9.56	9.51	9.55	17.22	16.98	17.21
11	12	100	60	8.14	8.05	8.13	13.13	13.00	13.14
12	12	100	40	8.26	8.19	8.25	14.34	14.12	14.33
13	8	80	40	8.14	8.10	8.13	15.12	15.25	15.13
14	16	80	40	7.55	7.56	7.56	14.01	14.01	14.02
15	12	80	50	9.67	9.67	9.66	16.48	16.51	16.47
(RSM optimization parameters)	8.46	63.23	49.49	9.66			16.98		
Experimental values				9.60 ± 0.25			16.91 ± 0.75		
% Difference				0.62			0.41		
(SVR optimization parameters)	9.28	65.43	49.61	9.81			17.19		
Experimental values				9.62 ± 0.28			16.88 ± 0.64		
% Difference				1.93			1.80		

X_1_, time (min); X_2_, amplitude (%); X_3_, temperature (°C); RSM, Response surface methodology; SVR, Support vector regression; FRAP, Ferric-reducing antioxidant power; TE, trolox equivalent.

**Table 2:** RSM regression parameters for total chlorophyll and FRAP under.

Source	DF	Total chlorophyll (g/100 mL)		FRAP (mmol TE/L)	
		*F*-Value	*P*-Value	*F*-Value	*P*-Value
Model	9	128.25	0.000	39.88	0.000
Linear	3	109.25	0.000	30.09	0.001
X_1_	1	180.73	0.000	37.58	0.002
X_2_	1	143.54	0.000	32.73	0.002
X_3_	1	3.48	0.121	19.97	0.007
Square	3	205.87	0.000	55.38	0.000
X_1_X_1_	1	503.50	0.000	47.38	0.001
X_2_X_2_	1	111.36	0.000	56.51	0.001
X_3_X_3_	1	68.89	0.000	87.01	0.000
2-way interaction	3	69.64	0.000	34.18	0.001
X_1_X_2_	1	180.50	0.000	101.60	0.000
X_1_X_3_	1	21.40	0.006	0.03	0.859
X_2_X_3_	1	7.01	0.046	0.91	0.383
Error	5				
Lack-of-fit	3	*	*	*	*
Pure error	2				
Total	14				
*R* ^2^		99.57%		98.63%	
Adj. *R*^2^		98.79%		96.15%	
Pred. *R*^2^		93.10%		78.02%	

X_1_, time; X_2_, amplitude; X_3_, temperature; DF, degrees of freedom; *R*^2^, coefficient of determination; FRAP, ferric-reducing antioxidant power; TE, trolox equivalent *p* < 0.05, significant differences; *p* < 0.01, very significant differences. *Does not matter in statistical calculation.

In the equation, Y denotes the dependent variable, *β*₀ is the intercept, βᵢ represents the linear coefficient, βᵢᵢ the quadratic coefficient, and βᵢⱼ the interaction coefficient between the independent variables X_i_ and X_j_.

Model adequacy was evaluated through ANOVA, lack-of-fit testing, *R*^2^, adjusted *R*^2^, predicted *R*^2^, and residual diagnostic plots. Significant model terms (*p* < 0.05) were retained for response surface generation. All statistical analyses and RSM procedures were performed using SPSS 22.0 software (SPSS Inc., Chicago, IL, USA).

#### 2.2.3 SVR modelling

Support Vector Regression (SVR) is a machine learning method that reduces overfitting and underfitting in modelling nonparametric experimental data based on kernel functions (18, 19). In our study, we used the RBF (Gaussian) kernel to model nonlinear relationships.

In this study, a 5-fold cross-validation was performed, and the hyperparameters (C, *ε*, *γ*) were optimised for the RBF kernel using GridSearchCV. The inputs were scaled using z-score standardization (mean = 0, std. = 1) with StandardScaler to compensate for kernel sensitivity. Data preprocessing is a critical step in using ML models, as it significantly affects model performance and accuracy (12). The mathematical representations of the RBF kernel (Equation 2) and the SVR prediction function (Equation 3) are given.


$$k(x_{i},x)=\exp(-\frac{\|x_{i}-x\|2}{2\sigma^{2}})$$ (2)

$$(x)=\sum_{i=1}^{n}(a_{i}^{*}-a_{i})k(x_{i},x)+b$$ (3)

In the modelling, the search space was defined as C ∈ {0.1, 1, 10, 100, 1,000}, *ε* ∈ {0.01, 0.1, 0.5, 1.0}, *γ* ∈ {“scale,” “auto,” 0.01, 0.1, 1}. The combination with the highest 5-fold CV average was selected, with *R*^2^ as the objective criterion. The final model was then retrained on the entire dataset. Predictions were made for all observations using the best models for each target variable (FRAP, CHLOROPHYLL).

#### 2.2.4 Determination of bioactive compounds

Total phenolic content (TPC) of rocket juice was determined using the Folin–Ciocalteu method, with results expressed as milligrams of gallic acid equivalents per litre (mg GAE/L) (20). The Folin–Ciocalteu reagent (Sigma-Aldrich, F9252) was used as supplied. The sodium carbonate solution (7.5% w/v) was prepared recently by dissolving 7.5 g of Na_2_CO_3_ (Merck, ≥99%) in 100 mL of distilled water. Reaction mixtures were incubated for 30 min in the dark at room temperature. Chlorophyll levels were measured spectrophotometrically according to the procedure of Hiscox and Israelstam (21). For this, 3 mL of rocket juice was mixed with an equal volume of 80% (v/v) acetone, and the mixture was filtered three times through Whatman filter paper to remove particulate matter. Absorbance of the final filtrate was recorded at 645 and 663 nm.

The total antioxidant capacity was evaluated using the FRAP assay, which is based on the reduction of Fe^3+^ to Fe^2+^ and formation of a colored complex measured at 593 nm. The FRAP reaction was initiated with a freshly prepared reagent mixture containing 300 mM acetate buffer (pH 3.6), 10 mM TPTZ solution (Sigma-Aldrich) in 40 mM HCl, and 20 mM FeCl_3_·6H_2_O, in a ratio of 10:1:1 (v/v/v). The mixture was incubated at 37 °C for 10 min prior to use. Results were calculated using a Trolox standard curve and reported as mmol TE/L (22).

Total carotenoid content was estimated using a modified spectrophotometric procedure (23, 24). In brief, 1 mL of rocket juice was mixed with 5 mL of methanol. After phase separation, the upper phase was collected. Saturated NaCl and a small amount of sodium sulphate were then added, after which the samples were centrifuged at 4,000 rpm for 10 min using a GYROZEN 1730 R centrifuge (Korea). The upper phase was then collected, diluted with methanol, and the absorbance was measured at 450 nm using a UV–VIS spectrophotometer (SP-UV/VIS-300SRB, Spectrum Instruments, Australia). Concentrations were determined using a *β*-carotene calibration curve and expressed as milligrams of β-carotene per litre (mg β-carotene/L).

#### 2.2.5 Volatile aroma compounds

Before analysis, 5 μL of cyclohexanone was added to each sample as an internal standard. Volatile compounds were extracted using headspace solid-phase microextraction (HS-SPME) with 50/30 μm DVB/CAR/PDMS fibres (Supelco, USA). To this end, 10 mL of the sample was placed in 22 mL sealed vials and pre-incubated at 50 °C for 10 min; the volatiles were then adsorbed at the same temperature for a further 20 min. Desorption was conducted at 250 °C for 1 min. Volatiles were separated using an Agilent 7,890 gas chromatograph equipped with a DB-5 column and a 5,977 N mass spectrometer. Helium was used as the carrier gas at a flow rate of 1.2 mL/min. Compound identification was achieved by matching mass spectra to entries in the NIST/EPA/NIH library, and semi-quantitative analysis was performed using the internal standard method.

#### 2.2.6 Phenolic profile

Phenolic compounds were analyzed using reversed-phase high-performance liquid chromatography (RP-HPLC) coupled with UV–Vis detection. Samples were extracted with ethyl acetate, kept in the dark, and concentrated by solvent evaporation. Analyses were performed on a 250 × 4.6 mm C18 column (5 μm) at 30 °C, using a linear gradient of phosphoric acid in water and acetonitrile as the mobile phase. Sample injection volume was 10 μL, and phenolics were monitored at 280, 320, and 360 nm. Identification and quantification were performed using commercial standards, with results reported as the mean of three replicate measurements. All phenolic models, comprising 98% chlorogenic acid, 99% caffeic acid, and 98% quercetin, were obtained from Sigma-Aldrich (St. Louis, USA). Stock solutions were formulated in methanol (HPLC grade, Merck) and preserved at −20 °C until analysis. Method validation was supported by calibration curves and bioinformatics-based data processing (25).

**Figure 1:**
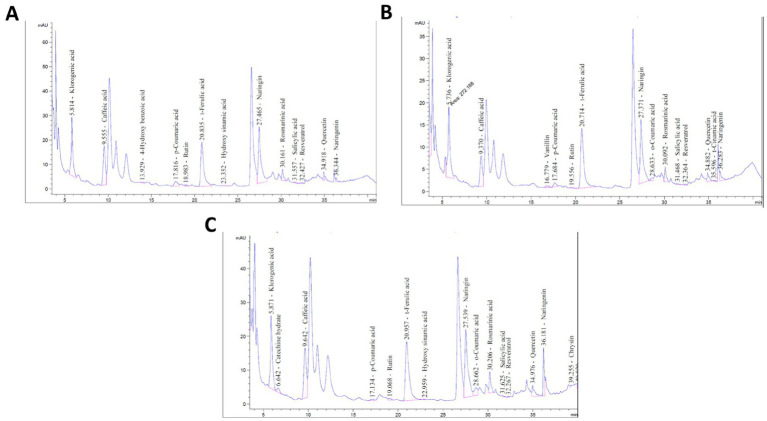
HPLC Chromatographic profiles of phenolic compounds in **(A)** (C-RJ), **(B)** (P-RJ) and **(C)** (TS-RJ) samples. C-RJ, Control rocket juice; P-RJ, thermal pasteurized rocket juice; TS-RJ, thermosonication-treated rocket juice.

Figure 1 shows the chromatograph of phenolic compounds (HPLC).

#### 2.2.7 Simulated gastrointestinal digestion *in vitro*

In this study, the in vitro digestion protocol developed by Minekus et al. was applied, followed by dialysis. The process consists of three consecutive stages: In the oral stage, samples were mixed with simulated saliva containing *α*-amylase (75 U/mL) at pH 7.0 and incubated for 2 min at 37 °C with gentle shaking. In the gastric stage, pepsin (2,000 U/mL) was added, the pH was adjusted to 3.0, and the samples were incubated for 2 h at 37 °C with continuous shaking. In the intestinal stage, pancreatin (100 U/mL) and bile salts (10 mM) were added, the pH was adjusted to 7.0, and the samples were incubated for 2 h at 37 °C. Following digestion, samples were centrifuged at 5,000×*g* for 10 min, and the supernatants were collected for further analysis. Following the gastrointestinal stages, samples were analyzed for total chlorophyll, TPC, and antioxidant capacity using the FRAP method. Triplicate measurements were performed for each trial and replicate, and mean values were reported (26).

#### 2.2.8 Statistical analysis

The experiments were repeated three times for each condition, and the results obtained are presented as mean values with standard deviation (SD). One-way analysis of variance (ANOVA) was used for statistical analysis, and the Tukey HSD (Honest Significant Difference) test was employed to determine significant differences between groups at the *p* < 0.05 level. All statistical procedures were performed using SPSS 22.0 software (SPSS Inc., Chicago, IL, USA).

## 3 Results and discussion

### 3.1 Effects of processing parameters on functional activity and bioactive components

In this study, data from a three-factorial experimental design were used to compare the RSM and SVR approaches for modelling FRAP and chlorophyll responses. The effect of thermosonication on total chlorophyll (Equation 4) and FRAP (Equation 5) is shown below.


$$\begin{array}{l} \text{Total Chlorophyll}\;(g/100\;\mathrm{mL})=-18.34+1.381\;X_{1}\\ +0.1445\;X_{2}+0.6196\;X_{3}-0.08062\;X_{1}X_{1}\\ -0.001517\;X_{2}X_{2}-0.004771\;X_{3}X_{3}+0.009275\;X_{1}X_{2}\\ -0.00639\;X_{1}X_{3}-0.000731 \end{array}$$ (4)

$$\begin{array}{l} \text{FRAP}\;(\text{mmol}\;\mathrm{TE}/L)=-22.11-0.089\;X_{1}+0.2258\;X_{2}\\ +1.327\;X_{3}-0.06112\;X_{1}X_{1}-0.002670\;X_{2}X_{2}\\ -0.01325\;X_{3}X_{3}+0.01720\;X_{1}X_{2}+0.00064\;X_{1}X_{3}\\ -0.000653\;X_{2}X_{3}\; \end{array}$$ (5)

The increase in X_1_ (time) and X_2_ (amplitude) positively affects the total chlorophyll value in rocket juice. This increase in total chlorophyll level can be associated with the acoustic and hydrodynamic cavitation phenomenon that occurs suddenly in the solvent environment, releasing high energy after expanding and collapsing (27, 28). Pooja et al. reported that prolonging ultrasound application time increased the total chlorophyll content of pea pods. This finding is similar to the results of our study (29).

Table 2 presents the ANOVA results. Accordingly, the RSM models for FRAP and total chlorophyll responses under thermosonication conditions are highly significant (*p* < 0.001). The *R*^2^ values, which indicate the model’s explanatory power, were 99.57% for total chlorophyll and 98.63% for FRAP, indicating a very high level of fit. Adjusted *R*^2^ (Adj. *R*^2^) values were 98.79 and 96.15%, respectively, indicating a low risk of overfitting the models.

Pred. *R*^2^ values, which indicate predictive power, were 93.10% for chlorophyll and 78.02% for FRAP. When linear terms were examined, it was observed that factors X_1_ (time) and X_2_ (amplitude) had statistically significant effects on both responses (*p* < 0.01). X_3_ (temperature) did not show a substantial impact on total chlorophyll (*p* > 0.05), but was a considerable variable for FRAP (*p* < 0.01). All square terms (X_1_^2^, X_2_^2^, X_3_^2^) were found to be highly significant for both responses (*p* < 0.01), supporting the nonlinear trend of the response variables and the existence of optimum conditions.

When interaction terms were evaluated, it was determined that the X_1_ × X_2_ combination had a strong, synergistic effect on both total chlorophyll and FRAP (*p* < 0.001). In contrast, the X_1_ × X_3_ interaction was only significant for chlorophyll (*p* < 0.01), while the X_2_ × X_3_ interaction was only marginally significant for chlorophyll (*p* < 0.05) and insignificant for FRAP (p > 0.05). Overall, these results suggest that chlorophyll extraction in thermosonication is primarily affected by time and amplitude parameters. In contrast, FRAP values are affected by the combination of time, amplitude, and temperature.

### 3.2 Results of the SVR

The SVR method with RSM was used to predict the FRAP and chlorophyll reactions. SVR is a robust ML algorithm for interpreting experimental data, as it can model non-linear relationships in high-dimensional spaces using kernel functions. The RBF (Gaussian) kernel was chosen for this modelling study. During the modelling process, the inputs were scaled using StandardScaler, and then the hyperparameters (C, *ε*, *γ*) were optimised by 5-fold cross-validation using GridSearchCV. The results obtained are summarised in Table 3. The model’s validation success for FRAP was high (CV *R*^2^ = 0.84), and an almost perfect fit (*R*^2^ ≈ 1.0, RMSE = 0.0101) to the training data was achieved. Although the validation performance for chlorophyll remained moderate (CV *R*^2^ = 0.66), a very high agreement with the training data was also observed (*R*^2^ = 0.999, RMSE = 0.0325). This indicates that the SVR performs well for FRAP predictions but may show partial overfitting for chlorophyll.

**Table 3:** Hyperparameters and performance metrics of the SVR models (RBF kernel) for FRAP and chlorophyll.

Target	Best CV *R*^2^	Train *R*^2^	Train RMSE	Best hyperparameters
FRAP	0.841	0.9999	0.0101	*C* = 100, *ε* = 0.01, *γ* = 0.1, kernel = rbf
Chlorophyll	0.666	0.9989	0.0325	*C* = 1,000, *ε* = 0.01, *γ* = 0.01, kernel = rbf

**Figure 2:**
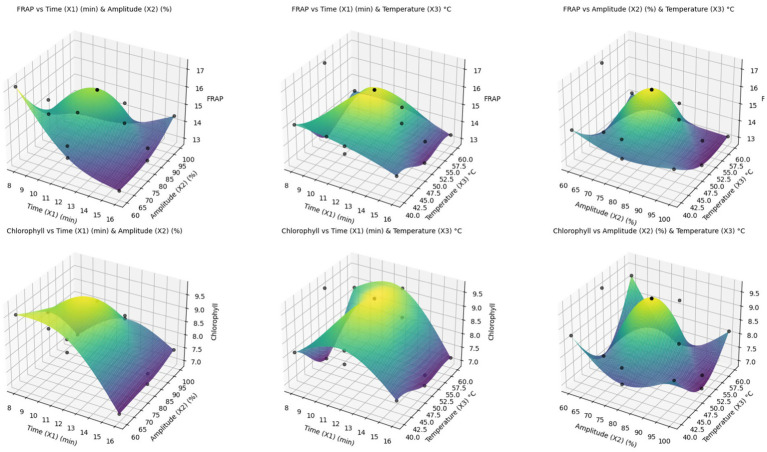
3D response surfaces based on a three-factor experimental design for FRAP and chlorophyll reactions.

Figure 2 shows the changes in FRAP and chlorophyll responses to three different factor combinations (time, amplitude, and temperature) using three-dimensional response surfaces. The upper surfaces belong to FRAP, while the lower surfaces belong to chlorophyll. Examining the figures, it is evident how the responses evolve as the other two factors change, while the two remaining factors remain constant. While a clear trend of curvature in the FRAP response can be observed, chlorophyll shows a relatively complex response pattern. Such surfaces contribute to a more precise evaluation of the interactions between the experimental factors and to the determination of the optimal conditions.

**Figure 3:**
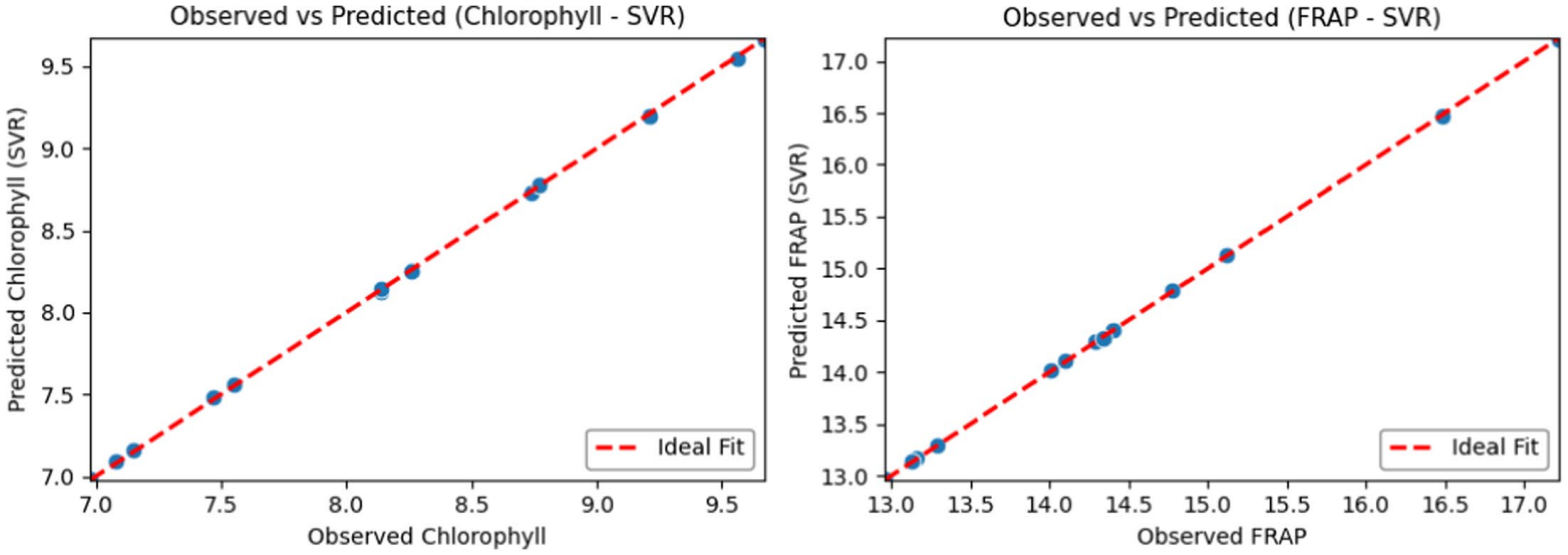
Observed–predicted scatter plots from SVR models: **(A)** Chlorophyll and **(B)** FRAP. The dashed line indicates the ideal 1:1 fit.

The performance of the SVR models was also visually confirmed using observed–predicted scatter plots for chlorophyll and FRAP (Figure 3). For both targets, clustering of points around the 1:1 ideal-fit line indicates that the model predictions are generally well calibrated. For FRAP, the tighter clustering along the line suggests higher predictive accuracy, while the relatively wider spread for chlorophyll suggests minor systematic deviations in a few cases. Nevertheless, no pronounced outliers or systematic trends were observed for either model; the plots support that the SVR approach achieves a robust fit across the experimental range.

**Figure 4:**
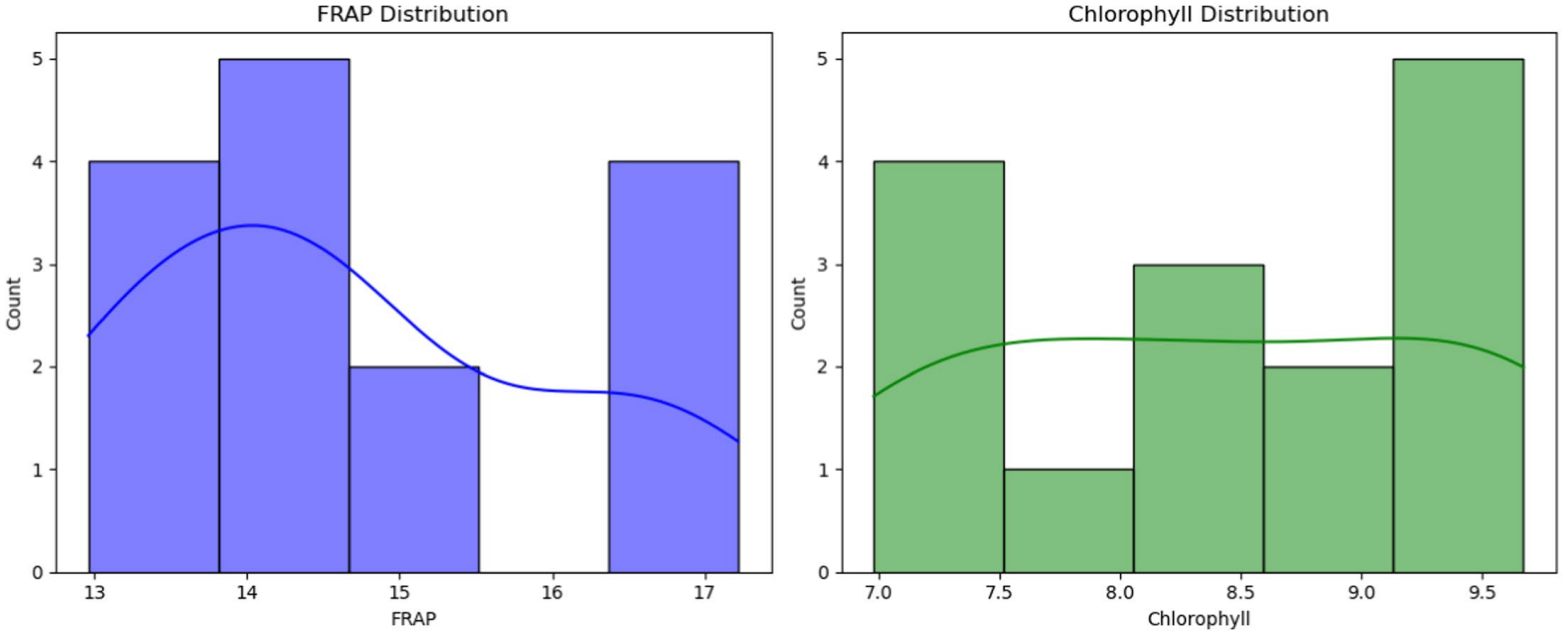
Histograms with kernel density estimates (KDE) for the response variables: **(A)** FRAP and **(B)** Chlorophyll. The panels show the empirical distributions of observed values with smooth KDE overlays highlighting central tendency and spread; both variables appear approximately unimodal with no pronounced outliers.

To examine the distributional characteristics of the response variables, histograms with kernel density estimates (KDE) were plotted for FRAP and Chlorophyll in Figure 4. Both distributions appear approximately unimodal with no pronounced outliers, suggesting that the observed values are well concentrated within the experimental range. The smooth KDE curves provide a visual check of symmetry and spread, which is helpful given the small sample size; notably, FRAP shows slightly less dispersion than chlorophyll. These distributional diagnostics complement the SVR results by indicating that the targets are well behaved and free of extreme values that could unduly influence model fitting.

The SVR models have proven effective at predicting experimental data. The high accuracy and low error values for FRAP indicate that this method is a powerful tool for predicting response variables. Although the validation performance for chlorophyll is relatively low, SVR’s ability to capture complex, nonlinear relationships complements and is essential when evaluated alongside RSM. Thus, it can be said that SVR is a powerful modelling approach for understanding interactions among different factors and determining optimal process conditions.

### 3.3 Total bioactive compound

**Figure 5:**
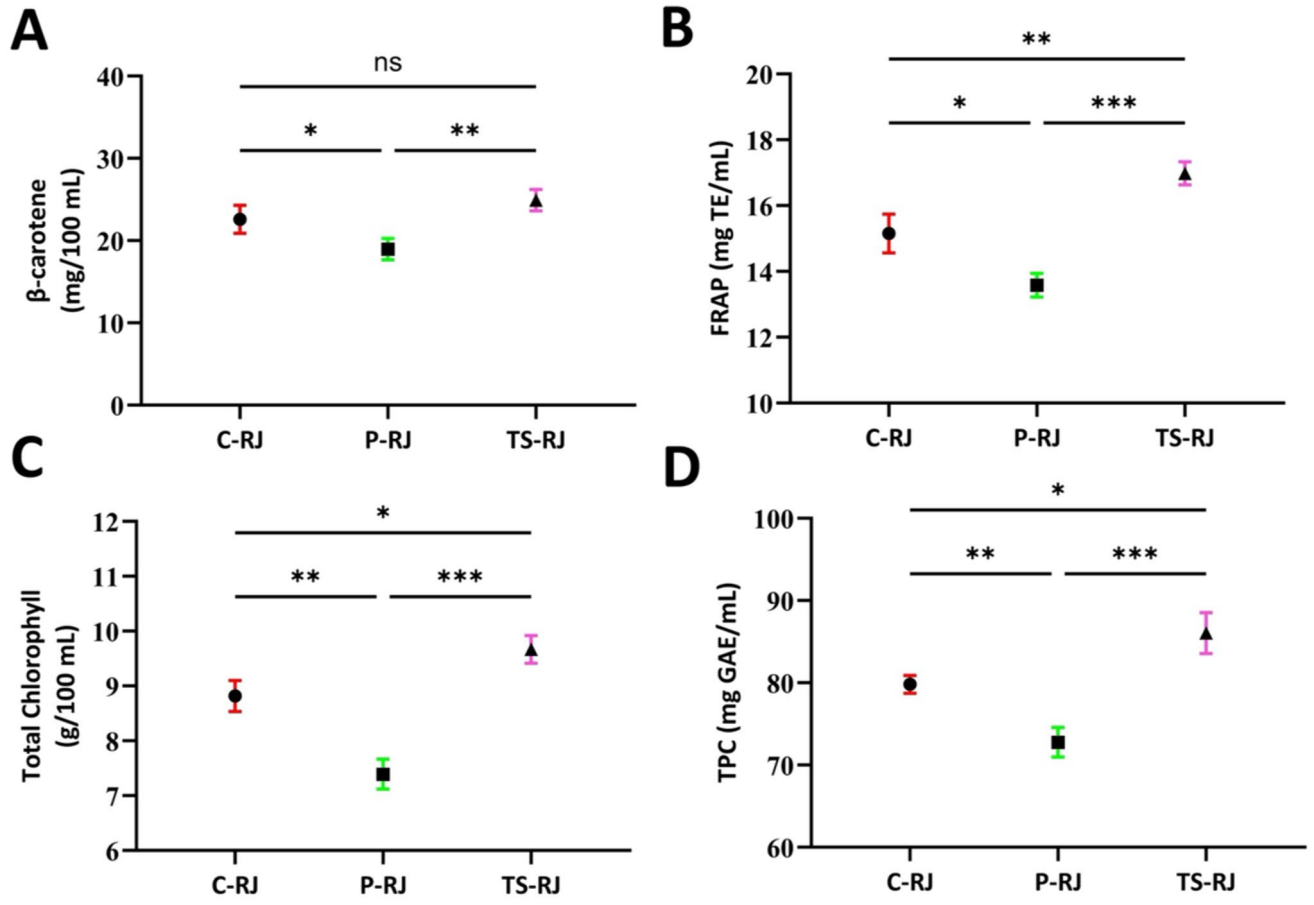
Comparison of bioactive components in rocket juice samples: **(A)** *β*-carotene, **(B)** FRAP, **(C)** total chlorophyll and **(D)** total phenolic content (TPC). C-RJ, Control rocket juice; P-RJ, Pasteurized rocket juice; TS-RJ, Thermosonicated rocket juice; FRAP, Ferric reducing antioxidant power; TPC, Total phenolic content; mg TE, Milligram trolox equivalent; mg GAE, Milligram gallic acid equivalent.

Figure 5 clearly demonstrates the effects of different treatment types on the bioactive components of rocket juice. Significant decreases (*p* < 0.05) were recorded in TPC (72.74 mg GAE/mL), total chlorophyll (7.39 g/100 mL), FRAP value (13.58 mg TE/mL), and *β*-carotene (18.95 mg/100 mL) following pasteurization. This suggests that high temperatures lead to oxidative degradation of phenolic compounds and structural degradation of pigments. In contrast, thermosonication demonstrated a protective and even enhancing effect on both phenolic compounds and pigments. The increase in bioactive compounds resulting from thermosonication is attributed to the cavitation phenomenon (30). Indeed, TPC (86.04 mg GAE/mL) and β-carotene (24.90 mg/100 mL) values were found to be above those in the control group, while total chlorophyll (9.67 g/100 mL) and FRAP (16.98 mg TE/mL) levels also showed significant increases (*p* < 0.05). The results show that thermosonication increases the release of biologically active components by breaking down cell walls and thereby improves the product’s functional properties. Similarly, various studies have reported that ultrasonic applications effectively increase the concentration of bioactive components in fruit juice and vinegar samples (31–33). Consistent with the obtained bioactive compound data, UV-C irradiation treatment led to an increase in antioxidant capacity in grape vinegar (34) and apple juice (35).

**Figure 6:**
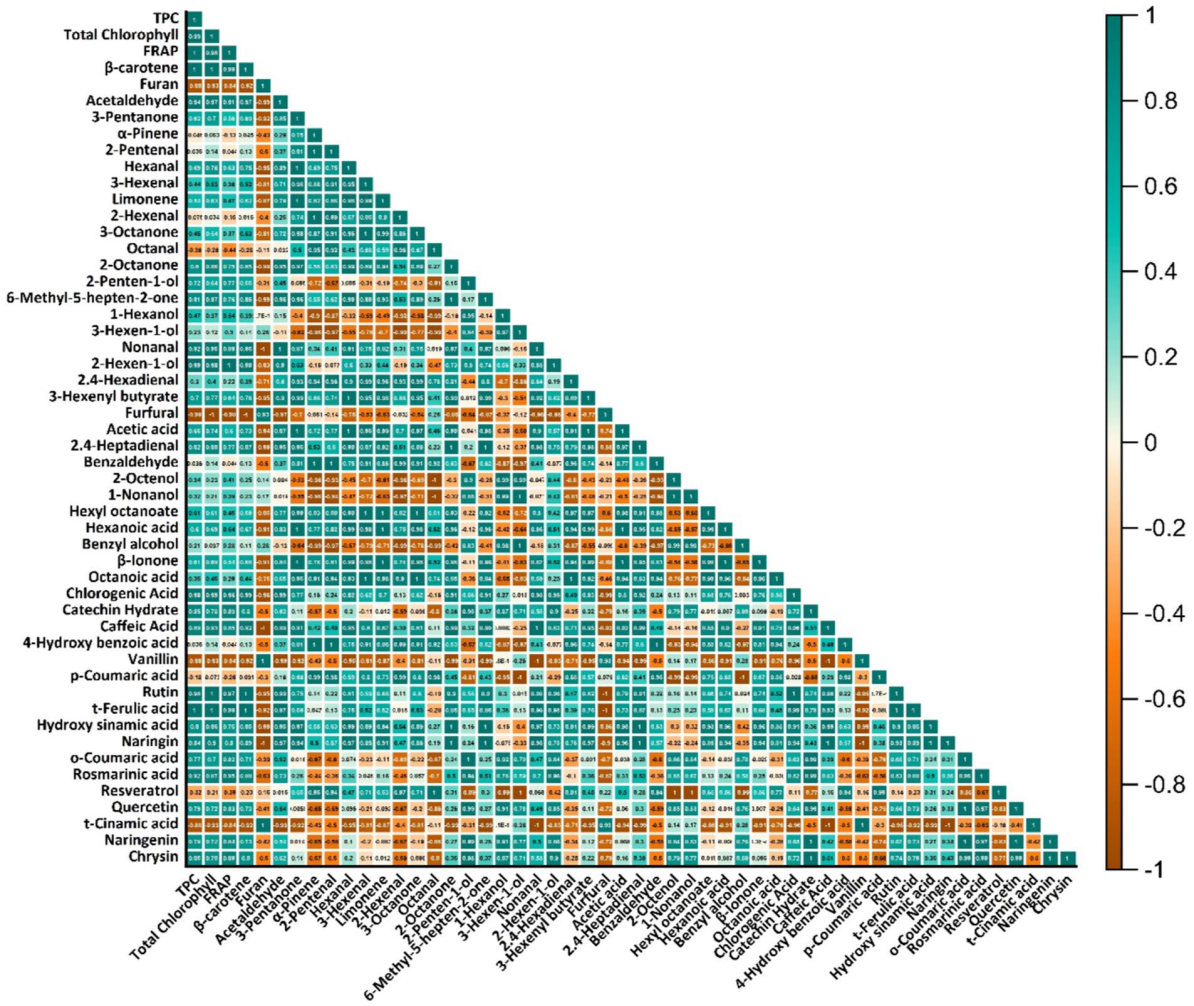
Pearson correlation heatmap illustrating relationships between bioactive parameters, phenolic compounds, and volatile aroma components.

The Pearson correlation heat map in Figure 6 provides a comprehensive assessment of interactions among bioactive compounds, phenolic profiles, and aroma compounds. Additionally, a positive correlation was observed between β-carotene and total chlorophyll (*r* = 0.72, *p* < 0.05). This result suggests that pigments are affected together due to their similar oxidative and photosensitivity properties. Furthermore, some aroma compounds showed moderate correlations with phenolic compounds (*r* ≈ 0.55–0.68, *p* < 0.05), indicating that sensory quality and functional properties change in parallel. Therefore, thermosonication was statistically superior not only in preserving the bioactive components but also in enhancing the synergy between functional quality and sensory profile.

### 3.4 Phenolic profile

Phenolic compounds are commonly found in plant-based foods. They have become the focus of increasing research interest. This is in the fields of nutrition and food sciences. This is due to their biological functions and health-beneficial properties (36).

When the phenolic profiles in Table 4 and the Pearson results presented in Figure 6 are evaluated together, it becomes apparent that the treatment types have a significant effect on the preservation of phenolic patterns and the studied relationships. It is generally observed that thermosonication treatments improve the phenolic profile, whereas thermal pasteurization treatments decrease its content. The findings suggest that thermal processing may degrade phenolic compounds through oxidation and heat-induced changes in flexibility. By contrast, thermosonication has been observed to increase the release of phenolic compounds bound to cell walls, while preserving or even enhancing the phenolic profile by minimising oxidation (37).

**Table 4:** Effect of various treatments on total phenolic compounds in rocket juice (C-RJ, P-RJ, TS-RJ).

Phenolic compounds (μg/mL)	Samples		
	C-RJ	P-RJ	TS-RJ
Chlorogenic acid	39.98 ± 2.36^ab^	34.73 ± 0.96^a^	42.05 ± 0.88^b^
Catechin hydrate	0.00 ± 0.00^a^	0.00 ± 0.00^a^	3.52 ± 0.15^b^
Caffeic acid	15.6 ± 0.92^b^	7.41 ± 0.21^a^	15.66 ± 0.68^b^
4-hydroxy benzoic acid	0.30 ± 0.02^b^	0.00 ± 0.00^a^	0.00 ± 0.00^a^
Vanillin	0.00 ± 0.00^a^	0.44 ± 0.01^b^	0.00 ± 0.00^a^
p-Coumaric acid	2.70 ± 0.18^c^	0.69 ± 0.03^b^	0.10 ± 0.00^a^
Rutin	0.54 ± 0.04^b^	0.00 ± 0.00^a^	0.78 ± 0.04^c^
t-Ferulic acid	29.69 ± 1.98^ab^	24.07 ± 0.67^a^	33.24 ± 1.70^b^
Hydroxy sinamic acid	0.60 ± 0.04^b^	0.00 ± 0.00^a^	0.50 ± 0.03^b^
Naringin	93.98 ± 6.26^b^	63.79 ± 1.77^a^	91.34 ± 4.69^b^
o-Coumaric acid	0.00 ± 0.00^a^	0.35 ± 0.01^a^	2.58 ± 0.15^b^
Rosmarinic acid	3.19 ± 0.16^a^	2.82 ± 0.08^a^	5.09 ± 0.15^b^
Salicylic acid	n.d.	n.d.	n.d.
Resveratrol	0.16 ± 0.01^c^	0.07 ± 0.00^b^	0.02 ± 0.00^a^
Quercetin	2.34 ± 0.14^a^	2.56 ± 0.07^a^	4.28 ± 0.25^b^
t-Cinamic acid	0.00 ± 0.00^a^	0.01 ± 0.00^b^	0.00 ± 0.00^a^
Naringenin	1.51 ± 0.09^a^	2.35 ± 0.07^a^	9.54 ± 0.56^b^
Chrysin	0.00 ± 0.00^a^	0.00 ± 0.00^a^	3.22 ± 0.19^b^

C-RJ, Control rocket juice; P-RJ, thermal pasteurized rocket juice; TS-RJ, thermosonication-treated rocket juice; n.d., could not be detected.

Compared to the control group, thermosonication resulted in a significant increase in the concentrations of chlorogenic acid (42.05 μg/mL), caffeic acid (15.66 μg/mL), and t-ferulic acid (33.24 μg/mL) (*p* < 0.05). In contrast, pasteurization was found to cause losses ranging from 15 to 50% in these compounds. In a study conducted by Dülger Altiner et al., it was reported that the chlorogenic acid (355.71 μg/mL) value increased statistically significantly after thermosonication applied to purple onion juice, similar to our study (9).

Correlation analysis revealed strong positive correlations between total phenolic content and these three hydroxycinnamic acids (*r* = 0.78–0.84, *p* < 0.01). This suggests that phenolics make a significant contribution to antioxidant capacity, and this contribution is particularly enhanced during the TS process.

When flavonoids were examined, thermosonication resulted in significant improvements in the levels of quercetin (4.28 μg/mL, ≈83% increase), naringenin (9.54 μg/mL, ≈6-fold increase), and rutin (0.78 μg/mL, ≈44% increase). Yıkmış et al. conducted a study using thermosonication on pollen-enriched uruset apple juice. This study observed significant improvements in the phenolic compound content of apple juices, including chlorogenic acid, quercetin, and catechin, following thermosonication compared to the control group. These results are consistent with those of our study (38). Pearson analysis also supports this finding, with moderate to high correlations (*r* = 0.62–0.75, *p* < 0.05) observed between flavonoid levels and antioxidant capacity. In another study, similar to our findings, after thermosonication of black carrot juice, the chlorogenic acid concentration was higher (149.26 μg/mL) in the thermosonicated sample than in the control sample (39).

Some aroma compounds, such as 3-Hexen-1-ol and 3-Hexenal, also showed positive correlations with quercetin and naringin (*r* ≈ 0.55–0.68, *p* < 0.05). This result suggests that thermosonication not only contributes to the preservation of phenolic compounds but also enhances the synergy between aroma profile and biological functionality (Table 4, Figure 6). However, some exceptions are noteworthy. Resveratrol decreased significantly in the TS treatment (0.16 → 0.02 μg/mL, ↓ ≈ 88%), while p-coumaric acid decreased in both treatments, reaching its lowest level in TS (0.10 μg/mL). Correlation analysis revealed weak negative correlations between these compounds and total phenolic content (*r* = −0.30 to −0.45), suggesting the involvement of different biochemical reactions. In conclusion, Table 4 and Figure 6 show that thermosonication generally provides a statistically significant advantage (*p* < 0.05) in preserving phenolic compounds and functional properties, but some phenolics (e.g., resveratrol, p-coumaric acid) are sensitive to processing conditions and require optimization.

### 3.5 Volatile aroma compounds

The analyses identified 31 aroma compounds in the rocket water samples, comprising 10 aldehydes, 5 ketones, 3 acids, 2 terpenes, 2 esters, 7 alcohols, and 2 other compounds. The highest amount of aroma compounds was 49.05 μg/kg in the C-RJ sample, 39.32 μg/kg in the P-RJ sample, and 47.99 μg/kg in the TS-RJ sample, respectively. The most common smell in rocket juice was 3-hexen-1-ol, 3-hexenal, limonene, 2-hexenal, and 3-hexenyl butyrate. The results reported by Jirovetz et al. and Arctander were similar (40, 41). When the aroma compounds listed in Table 3 are evaluated, it is evident that the applied processing methods have significantly altered the volatile profile of rocket juice. Characteristic compounds, such as 3-Hexenal (12.08 ± 0.54 μg/kg) and Limonene (3.29 ± 0.24 μg/kg), which were detected at high levels in the control group (C-RJ), decreased significantly after pasteurization (*p* < 0.05). Similarly, decreases were observed in compounds such as 2.4-Hexadienal and 3-Hexenyl butyrate due to pasteurization. This may be due to oxidative degradation due to high temperatures. Some compounds, such as furan, were detected only in the pasteurized sample, suggesting that this treatment produces new aroma compounds specific to heat treatments such as the Maillard reaction. The same results were reported by Cheng et al. (42) and Yıkmış et al. (43). Thermosonication treatment (TS-RJ) was determined to have a more protective effect on aroma compounds. In particular, alcohols such as 3-Hexen-1-ol (15.70 ± 1.18 μg/kg), 1-Hexanol (2.01 ± 0.23 μg/kg), and Benzyl alcohol (1.89 ± 0.17 μg/kg) showed higher levels than in the control and pasteurized samples. Furthermore, the increase in 2-octanol (1.02 ± 0.09 μg/kg) following thermosonication suggests that this method may also lead to the formation of new compounds through controlled cavitation. Two terpenes, Limonene and *α*-pinene, were detected in all rocket juice samples. In a study examining the impact of temperature abuse and improper packaging on the volatile profile of rocket leaves by Mastrandrea et al. (44), it was determined that Limonene and α-pinene were the dominant terpene compounds. Limonene was the most abundant terpenoid compound (1.82–3.29 μg/kg) in all rocket juice samples. Statistically significant differences in some compounds were not associated with treatment type, as indicated in the table. For example, compounds such as octanal (0.37–0.42 μg/kg), 2-octanone (0.21–0.33 μg/kg), and *β*-ionone (0.07–0.12 μg/kg) were detected at similar levels across all three treatment groups, suggesting that these volatile compounds were relatively stable. However, pasteurization clearly had adverse effects, particularly on aldehydes (e.g., hexanal, 2,4-heptadienal), while thermosonication minimized these losses while increasing the levels of some volatile compounds. These results support the industrial-scale applicability of non-thermal technologies for maintaining aroma stability (Table 5).

**Table 5:** Effect of different treatments on total aroma components in rocket juice (C-RJ, P-RJ, TS-RJ).

Aroma components	Samples		
	C-RJ	P-RJ	TS-RJ
Furan (802)	0.00 ± 0.00^a^	0.14 ± 0.02^b^	0.00 ± 0.00^a^
Acetaldehyde (821)	0.16 ± 0.03^b^	0.00 ± 0.00^a^	0.19 ± 0.04^b^
3-Pentanone (978)	0.66 ± 0.11^b^	0.24 ± 0.02^a^	0.49 ± 0.05^ab^
α-Pinene (1,025)	0.24 ± 0.02^a^	0.14 ± 0.04^a^	0.13 ± 0.03^a^
2-Pentenal (1,076)	0.18 ± 0.01^b^	0.00 ± 0.00^a^	0.00 ± 0.00^a^
Hexanal (1,084)	0.61 ± 0.13^a^	0.21 ± 0.06^a^	0.48 ± 0.06^a^
3-Hexenal (1,156)	12.08 ± 0.54^b^	6.24 ± 0.39^a^	8.62 ± 0.92^a^
Limonene (1,191)	3.29 ± 0.24^b^	1.82 ± 0.19^a^	2.57 ± 0.21^ab^
2-Hexenal (1,227)	3.24 ± 0.22^a^	2.66 ± 0.28^a^	2.58 ± 0.22^a^
3-Octanone (1,258)	0.24 ± 0.04^a^	0.12 ± 0.01^a^	0.17 ± 0.04^a^
Octanal (1,290)	0.42 ± 0.13^a^	0.39 ± 0.08^a^	0.37 ± 0.03^a^
2-Octanone (1,302)	0.33 ± 0.11^a^	0.21 ± 0.06^a^	0.31 ± 0.06^a^
2-Penten-1-ol (1,320)	0.41 ± 0.08^a^	0.47 ± 0.03^a^	0.69 ± 0.09^a^
6-Methyl-5-hepten-2-one (1,341)	0.13 ± 0.01^b^	0.00 ± 0.00^a^	0.11 ± 0.01^b^
1-Hexanol (1,356)	1.77 ± 0.34^a^	1.89 ± 0.13^a^	2.01 ± 0.23^a^
3-Hexen-1-ol (1,382)	11.17 ± 1.29^a^	14.48 ± 0.89^a^	15.7 ± 1.18^a^
Nonanal (1,396)	0.52 ± 0.15^a^	0.18 ± 0.06^a^	0.56 ± 0.09^a^
2-Hexen-1-ol (1,406)	0.91 ± 0.25^a^	0.78 ± 0.16^a^	1.08 ± 0.19^a^
2.4-Hexadienal (1,416)	3.09 ± 0.52^a^	1.52 ± 0.33^a^	1.95 ± 0.28^a^
3-Hexenyl butyrate (1,462)	2.73 ± 0.41^a^	1.63 ± 0.11^a^	2.39 ± 0.18^a^
Furfural (1,465)	0.48 ± 0.12^a^	0.63 ± 0.17^a^	0.39 ± 0.11^a^
Acetic acid (1,469)	0.14 ± 0.04^a^	0.00 ± 0.00^a^	0.09 ± 0.04^a^
2.4-Heptadienal (1,471)	1.06 ± 0.33^a^	0.44 ± 0.10^a^	0.98 ± 0.06^a^
Benzaldehyde (1,541)	0.23 ± 0.06^b^	0.00 ± 0.00^a^	0.00 ± 0.00^a^
2-Octenol (1,632)	0.46 ± 0.11^a^	0.81 ± 0.08^ab^	1.02 ± 0.09^b^
1-Nonanol (1,661)	1.46 ± 0.27^a^	1.77 ± 0.18^a^	1.94 ± 0.06^a^
Hexyl octanoate (1,796)	0.69 ± 0.04^a^	0.38 ± 0.06^a^	0.53 ± 0.13^a^
Hexanoic acid (1,850)	0.33 ± 0.10^b^	0.00 ± 0.00^a^	0.19 ± 0.04^ab^
Benzyl alcohol (1,882)	1.29 ± 0.15^a^	1.74 ± 0.11^a^	1.89 ± 0.17^a^
β-Ionone (1,959)	0.12 ± 0.04^a^	0.00 ± 0.00^a^	0.07 ± 0.03^a^
Octanoic acid (2,064)	0.61 ± 0.09^a^	0.43 ± 0.11^a^	0.49 ± 0.04^a^

C-RJ, Control rocket juice; P-RJ, thermal pasteurized rocket juice; TS-RJ, thermosonication-treated rocket juice.

### 3.6 Bioaccessibility and digestive stability

The effects of various treatments on rocket water were evaluated in terms of bioactive compound stability and bioavailability during *in vitro* digestion (Table 6). In the undigested phase, the highest values for chlorophyll, TPC, β-carotene and FRAP were observed in samples applied to thermosonication (TS-RJ). Total chlorophyll (9.67 ± 0.25 g/100 mL), FRAP (16.98 ± 0.35 mmol TE/L), TPC (86.04 ± 2.50 mg GAE/100 mL), and β-carotene (24.90 ± 1.30 mg/100 mL) levels were found to be significantly higher in the TS-RJ group compared to the control (C-RJ) and pasteurized (P-RJ) groups (*p* < 0.05). In pasteurized samples (P-RJ), lower chlorophyll (7.39 ± 0.27 g/100 mL) and *β*-carotene (19.85 ± 1.28 mg/100 mL) contents indicate thermal degradation and spoilage. The oral digestion phase resulted in a significant decrease in bioactive compounds across all groups. However, TPC (55.66 ± 1.60 mg GAE/100 mL) and total chlorophyll (6.25 ± 0.16 g/100 mL) values were significantly higher in the TS-RJ group than in the C-RJ and P-RJ groups. This suggests that thermosonication improves the preservation of phenolic compounds during the early stages of digestion. Thermal treatments applied to beetroot juice, particularly pasteurization, result in a significant decrease in antioxidant capacity and bioactive compounds, such as betacyanin, during the oral phase. While the reduction in betacyanin levels continued in the later stages of the digestive process, an increase in antioxidant capacity was observed in the gastric and intestinal phases. These findings suggest that pasteurization may have adverse effects on the stability and accessibility of bioactive compounds within the digestive tract (45). On the other hand, in another study comparing thermal treatments and thermosonication on beetroot juice, it was found that thermosonication not only preserved the antioxidant properties but also yielded better results in the in vitro digestion process (46). These examples align with the findings of our study, which support the notion that innovative non-thermal processes have significant potential for preserving bioactive compounds.

**Table 6:** Impact of treatments on bioactive compounds and antioxidant activity of rocket juice during simulated gastrointestinal digestion.

Phases	Samples	Total chlorophyll (g/100 mL)	FRAP (mmol TE/L)	TPC (mg GAE/100 mL)	β-carotene (mg/100 mL)
Undigested	C-RJ	8.82 ± 0.28^b^	15.15 ± 0.59^b^	79.80 ± 1.09^b^	22.59 ± 1.69^ab^
	P-RJ	7.39 ± 0.27^a^	13.58 ± 0.36^a^	72.74 ± 1.79^a^	18.95 ± 1.28^a^
	TS-RJ	9.67 ± 0.25^c^	16.98 ± 0.35^c^	86.04 ± 2.50^c^	24.90 ± 1.30^b^
Oral digestion	C-RJ	5.63 ± 0.18^b^	9.67 ± 0.38^b^	50.95 ± 0.69^b^	14.42 ± 1.08^b^
	P-RJ	4.59 ± 0.17^a^	8.33 ± 0.27^a^	45.21 ± 1.11^a^	11.80 ± 0.82^a^
	TS-RJ	6.25 ± 0.16^c^	10.85 ± 0.40^a^	55.66 ± 1.60^c^	16.11 ± 0.84^b^
Gastric digestion	C-RJ	4.27 ± 0.13^b^	7.30 ± 0.21^b^	38.71 ± 0.54^b^	10.94 ± 0.84^b^
	P-RJ	3.38 ± 0.16^a^	6.25 ± 0.16^a^	33.46 ± 0.83^a^	8.72 ± 0.59^a^
	TS-RJ	4.80 ± 0.11^c^	8.45 ± 0.16^c^	42.72 ± 1.38^c^	12.40 ± 0.65^b^
Intestinal digestion	C-RJ	2.78 ± 0.09^b^	4.78 ± 0.19^b^	21.50 ± 0.86^a^	6.89 ± 0.46^b^
	P-RJ	2.12 ± 0.17^a^	3.85 ± 0.17^a^	18.75 ± 2.34^a^	5.50 ± 0.38^a^
	TS-RJ	3.32 ± 0.14^c^	5.72 ± 0.16^c^	29.31 ± 1.89^b^	7.96 ± 0.23^c^
Recovery %	C-RJ	31.52 ± 0.04^ab^	31.54 ± 0.00^b^	26.94 ± 0.77^a^	30.54 ± 0.87^a^
	P-RJ	28.68 ± 2.11^a^	28.36 ± 1.08^a^	25.79 ± 3.38^a^	29.02 ± 0.08^a^
	TS-RJ	34.33 ± 0.92^b^	33.74 ± 1.62^b^	34.04 ± 1.41^b^	32.05 ± 2.13^a^

C-RJ, Control rocket juice; P-RJ, thermal pasteurized rocket juice; TS-RJ, thermosonication-treated rocket juice; TPC, Total phenolic content; mg GAE, milligram gallic acid equivalent; FRAP, Ferric reducing antioxidant power. Values are expressed as mean ± standard deviation (*n* = 3). Different superscript letters within the same digestion phase indicate significant differences between treatments (*p* < 0.05).

The decreasing trend continued in the gastric phase. The TS-RJ group maintained higher TPC (42.72 ± 1.38 mg GAE/100 mL) and FRAP (8.45 ± 0.16 mmol TE/L) levels than the control and pasteurized groups. Particularly in the pasteurized samples (P-RJ), the loss of both chlorophyll (3.38 ± 0.16 g/100 mL) and *β*-carotene (8.72 ± 0.59 mg/100 mL) was clearly noticeable. Bioactive compounds decreased to the lowest levels in all groups during the intestinal phase. However, the TS-RJ group distinguished itself by maintaining the highest TPC (29.31 ± 1.89 mg GAE/100 mL), FRAP (5.72 ± 0.16 mmol TE/L), and chlorophyll (3.32 ± 0.14 g/100 mL) values even in this phase. The C-RJ and P-RJ samples showed lower values in this phase. The samples from TS-RJ showed the highest recovery rates for chlorophyll (34.33%), FRAP (33.74%), and TPC (34.04%) when total recovery was evaluated. Control samples (C-RJ) were lower, particularly for TPC (26.94%). Recovery rates in pasteurized samples (TPC 25.79%, FRAP 28.36%) were also significantly lower than those in TS-RJ. In conclusion, thermosonication not only increased the initial amount of bioactive compounds but also enhanced their bioavailability by better preserving them throughout the digestive process. In contrast, pasteurization decreased the recovery rate by increasing the loss of bioactive compounds during the digestive phases. Even if fruit juices are not processed, the digestive process significantly reduces the bioaccessibility and antioxidant capacity of bioactive compounds (47). A study examining various thermal and non-thermal technologies on tropical fruit juices reveals that these compounds have the potential to maintain or increase their stability and bioaccessibility. Although the effects of treatments vary depending on fruit type and compound properties, both in our study and in similar studies, remarkable findings have been reported, especially in the development of innovative non-thermal methods (48).

## 4 Conclusion

This study has demonstrated that thermosonication significantly increases the levels of phenolic compounds, chlorophyll, β-carotene, and antioxidant capacity in arugula juice, providing superior protection of biologically active components compared to pasteurization. Significant increases in phenolics, such as chlorogenic acid, kuersetin, and narenine, have been recorded, particularly in terms of aroma compounds. It has been determined that compounds such as 3-Hexen-1-ol, 1-Hexanol, and Lemonene, which contribute to a fresh and characteristic profile, are preserved at high levels. *In vitro* digestive analyses have demonstrated that thermosonication not only increases the initial amount of compounds but also enhances their bioavailability throughout the digestive process. Statistically, RSM provides reliable results with high compliance coefficients, while the SVR model has emerged as a leading approach, particularly in estimating non-linear relationships and complex data structures. Therefore, the use of both approaches together provides more comprehensive results in process optimization. In general, it has been concluded that thermosonication is an innovative technology that maintains the functional quality of sensitive products, such as rocket water, increases bioavailability, and is highly applicable on an industrial scale.

## Data Availability

The original contributions presented in the study are included in the article/supplementary material, further inquiries can be directed to the corresponding author/s.
